# A mobile electrical stimulator for therapeutic modulation of the vestibular system — design, safety, and functionality

**DOI:** 10.3389/fneur.2024.1502204

**Published:** 2024-11-13

**Authors:** Sandra Kollmansperger, Julian Decker, Sebastian Berkes, Klaus Jahn, Max Wuehr

**Affiliations:** ^1^German Center for Vertigo and Balance Disorders (DSGZ), Ludwig-Maximilians-University of Munich, Munich, Germany; ^2^Schön Klinik Bad Aibling, Bad Aibling, Germany; ^3^neuroConn GmbH, Ilmenau, Germany; ^4^Department of Neurology, Ludwig-Maximilians-University of Munich, Munich, Germany

**Keywords:** galvanic vestibular stimulation, stochastic resonance, bilateral vestibulopathy, remote therapy, electrical stimulation, balance disorders, motion sensor

## Abstract

Low-intensity noisy galvanic vestibular stimulation (nGVS) is a promising non-invasive treatment for enhancing vestibular perceptual performance and postural control in patients with chronic vestibular hypofunction. However, this approach has so far been studied mainly under laboratory conditions. Evidence indicates that continuous application of nGVS in daily life is necessary for it to be effective. To address this need, we have developed a mobile nGVS stimulator and conducted a series of pilot studies to evaluate its safety, tolerability, functionality, and therapeutic effects. The device is a lightweight, compact, and portable AC stimulator featuring a user-friendly interface for the individualized adjustment of nGVS parameters. It includes an integrated motion sensor that automatically activates stimulation during body movement and deactivates it during inactivity, optimizing its practical use in real-world settings. The stimulator adheres to strict safety standards and, in initial long-term use, has exhibited only mild side effects (e.g., skin irritation and headaches), likely attributable to the current electrode placement, which requires further optimization. As expected, the device consistently elicits known vestibular sensorimotor reflex responses in healthy individuals. Importantly, further pilot studies in healthy participants demonstrate that the device can reliably replicate known facilitating effects on vestibular perception and postural control. Together, these findings suggest that this mobile stimulation device can facilitate the translation of nGVS into therapeutic everyday use.

## 1 Introduction

A permanent reduction of vestibular function can lead to symptoms such as postural instability or impaired gaze stabilization during head movements (1–3). These symptoms are particularly pronounced when affected individuals cannot compensate for the vestibular loss with other sensory inputs, such as while walking in the dark or on uneven surfaces (4, 5). As a result, this can significantly restrict everyday mobility, increase the risk of falls, and negatively affect quality of life (5–7). Chronic vestibular hypofunction occurs in well characterized cases of peripheral bilateral vestibulopathy (BVP) due to different aetiologies; it also becomes more common with aging due to the deterioration of peripheral vestibular structures (also called presbyvestibulopathy) (2, 8) and is sometimes associated with central neurodegenerative disorders, such as Parkinson’s disease (9).

Current therapeutic options for vestibular loss are largely limited to physical therapy, which aims to train the remaining intact sensory systems to compensate for the deficit. While this therapy is effective for most patients, it rarely leads to a sufficient improvement (10). Vibrotactile feedback is being explored to either enhance physical therapy (11) or provide continuous support for postural regulation in daily life (12). Besides, several alternative treatment approaches are being developed that aim to directly address vestibular hypofunction. One approach involves the use of a vestibular implant, which has shown promising effects in alleviating postural and other vestibular-related symptoms in selected patients (13). However, the benefits of such an invasive vestibular implant must be carefully balanced against the risks. A key limitation of such an implant is that it can only replace parts of the peripheral vestibular function, specifically the semicircular canals, but not the otolith organs (14, 15). Moreover, the precise placement of the implant is critical and involves several surgical risks, particularly the potential for permanent hearing loss.

Most patients with chronic vestibular hypofunction retain some residual vestibular function (1, 16). For these patients, a promising non-invasive alternative therapeutic approach involves enhancing this residual function through electrical low-intensity noise stimulation of the vestibular periphery, i.e., noisy galvanic vestibular stimulation (nGVS) (17, 18). A typical finding in patients with residual function is elevated detection thresholds for processing vestibular stimuli. Consequently, a relevant portion of naturally occurring vestibular stimuli remains subthreshold and therefore goes undetected (19). The idea behind the nGVS treatment approach is to lower these elevated thresholds through a phenomenon known as stochastic resonance, according to which the presence of a non-zero stochastic interference (i.e., noise) in a sensory system can amplify subthreshold stimuli and raise them above the detection threshold (20, 21). The weak noise in the vestibular periphery is achieved through galvanic vestibular stimulation (GVS) – an established technique for modulating vestibular receptors and afferents (22). In young healthy adults, nGVS lowers the vestibular perception threshold at intermediate, imperceptible noise levels (23–25) indicating that the signal-to-noise ratio in the vestibular system can be enhanced by weak external noise, even in this population. Beyond that, nGVS has been proven to facilitate a broad range of therapeutic effects in different clinical cohorts. In patients with BVP, it has been shown to enhance residual vestibular perceptual and sensorimotor functions while stabilizing balance during both static and dynamic postural tasks such as walking (26–30). Similar effects have been observed in elderly individuals and patients with various neurodegenerative diseases such as Parkinson’s disease (31–33) or Progressive Supranuclear Palsy (34).

The primary limitation of these experiments is their laboratory-based nature. The ecological validity of these treatment effects still needs to be evaluated in everyday applications with clinically relevant endpoints such as mobility, fall risk, and quality of life. Another significant factor is that most previous studies focused on short-term stimulation protocols below 1 h. While improvements in performance are frequently observed during stimulation, there is little evidence from previous studies to support long-lasting aftereffects of the stimulation (35, 36). Therefore, it is likely that continuous treatment with nGVS will be necessary to obtain sustained treatment effects. However, the feasibility and effectiveness of this approach in long-term use in everyday patient life remain unclear.

Evaluating the effects of nGVS in everyday life over longer periods requires a mobile and everyday-suitable stimulation device. In response to this need, we developed a prototype wearable nGVS device in collaboration with an industry partner (neuroConn GmbH, Ilmenau, Germany). In the following, we will present the fundamental technical requirements for the stimulator and the design of the prototype. Subsequently, we will discuss initial findings regarding the tolerability of long-term stimulation with the prototype and the occurrence of side effects. After that, we will present initial results that assess the function of the device (i.e., vestibular nature of stimulation), particularly in terms of its ability to elicit vestibular sensorimotor responses at suprathreshold intensities. In the final section, we will present a series of experiments that attempt to replicate previously known facilitatory effects on vestibular perception and postural control using the mobile therapeutic device.

## 2 Stimulator design

We identified a set of requirements that the mobile stimulation device must meet to be suitable for use in patients. Since it is intended for all-day use, it must be comfortably wearable, portable, and energy-efficient to facilitate full-day usage. As nGVS therapy is only needed when the patient is actively moving, the stimulation device should be activated by head or body movement associated with stance and gait and automatically deactivate after a period of inactivity. Finally, the device should include a simple user interface, as the stimulation parameters for nGVS are known to vary between patients and therefore should be manually adjustable.

**Figure 1:**
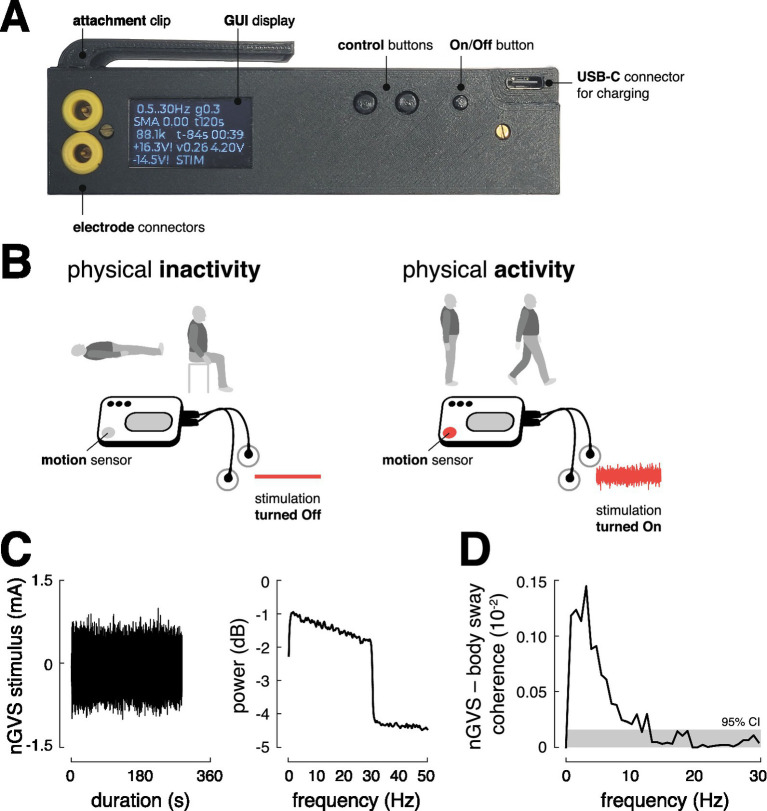
Stimulator design and function. **(A)** Prototype of the stimulator with control buttons and a display for individual adjustment of the stimulation mode and an external charging option via USB-C connector. **(B)** The mobile stimulator contains an integrated motion sensor that can selectively turn the stimulation on and off depending on the user’s activity or inactivity. **(C)** Profile of the nGVS stimulus and corresponding power spectrum. **(D)** Significant coupling between the nGVS stimulus (±1 mA peak amplitude) and body sway, as assessed via magnitude-squared coherence (the gray area indicates the 95% confidence interval), suggests that the nGVS stimulus at suprathreshold intensity activates vestibular sensorimotor pathways as intended. GUI, graphical user interface; nGVS, noisy galvanic vestibular stimulation; CI, confidence intervals.

To meet these requirements, we designed a compact and lightweight prototype device (L x W x H: 122 × 32 × 30 mm, 100 g) that is robust enough to withstand being dropped and water-resistant to protect against moisture and water splashes (Figure 1A). It is powered by a rechargeable Li-Ion battery (3.7 V, 3100mAh) providing a battery life of at least 24 h, considering a typical current consumption (i.e., 65 mA). The device includes a triaxial accelerometer (MPU-6050, TDK InvenSense, Tokyo, Japan) that continuously monitors the patient’s movement and automatically switches the stimulation on at defined thresholds (movement intensity >0.135 g (37)) (Figure 1B). The stimulation is automatically switched off after an adjustable period (6 s – 30 min) during which the stimulation device remains still. The device includes a simple user interface (screen and adjustment buttons) through which the stimulation intensity, minimum stimulation duration, and other settings can be adjusted, and information about the current stimulation mode, operating voltage, impedance, etc., can be viewed. The AC source generates a zero-mean white noise signal (0.5–30 Hz) with 10 adjustable peak amplitudes ranging from ±0.1 mA to ±1.0 mA.

To ensure the device’s output during stimulation is equivalent to other white noise-generating devices, the mean output current was measured using a digital multimeter (SDM3065X, Siglent Technologies, Shenzhen, China), and the spectral properties of the passband, as well as the signal-to-noise ratio, were analyzed with a digital oscilloscope (DHO1072, Rigol Technologies, Suzhou, China). These analyses confirmed that the prototype’s stimulation output is consistent with that of established stimulation devices. For all investigations reported below, the stimulator was attached to the collar of the participants and stimulation was delivered via a pair of conductive-rubber electrodes (40 × 60 mm) attached over the left and right mastoid process behind the ears. The electrodes were held in place using an elastic headband. Electrode gel was applied before electrode placement to achieve uniform current density and minimize any irritation to the skin due to stimulation.

To ensure not only user comfort but also safety, the design of the mobile nGVS device incorporates several critical features that balance usability with safety measures. Internal safety features of the nGVS device include automatic shut-off, overload protection, and gradual adjustments in stimulation intensity to prevent abrupt changes during stimulation on- or offset (2 s interval fade in/ fade out). The device limits the operating voltage to ±17 V through firmware restricted pulse width modulation (PWM) control and hardware constraints to ±18 V using Zener diodes. Output power is further limited by a restricted pulse width for the step-up DC/DC converters, also reducing voltage under unscheduled load conditions. Additionally, the device employs AC coupling to prevent the application of DC currents, eliminating the possibility of charge accumulation during stimulation. To protect both the device and the patient, the system employs a relay that diverts unwanted currents potentially occurring during non-stimulation. Additionally, the battery automatically shuts off in case of under-voltage. Finally, the maximum duration of continuous stimulation, when the device is not being moved, is set to 30 min.

## 3 Safety and tolerability of stimulation

**Figure 2:**
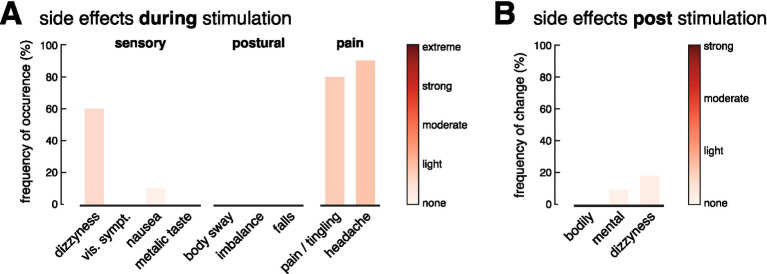
Safety and tolerability of stimulation. Frequency and intensity of side effects experienced during **(A)** and 10 min after **(B)** a long-term stimulation (2 h) with the mobile stimulation device. During stimulation, 60% of participants reported mild dizziness that resolved immediately after, while 90% experienced mild headaches and 80% reported tingling at the electrode sites. Adjusting electrodes reduced discomfort in most cases. No serious side effects were reported after the stimulation.

Based on these safety measures, we evaluated the safety and tolerability of a long-term application of the mobile nGVS device on 10 healthy individuals (5 females; mean age 28.7 ± 3.6 years). Participants were initially asked to complete a questionnaire assessing their current mental and physical condition (5-point Likert scale ranging from 0 (no symptoms/limitations) to 5 (strong symptoms/limitations)). Following this, participants wore the activated stimulator (at a subthreshold stimulation intensity set to 0.3 mA) for a period of 2 h. Participants were instructed to engage in their normal activities during the experiment, with some remaining stationary and others engaging in light physical activities (e.g., household tasks). After 1 h of stimulation, participants were asked to report their mental and physical condition (see Figure 2), with particular attention to any side effects and intensity [5-point Likert scale ranging from 0 (no symptoms/limitations) to 5 (strong symptoms/limitations)] commonly noted in previous studies on electrical stimulation (38). Ten minutes after the stimulation period, participants were again asked to report on their current mental and physical condition and regarding any noticeable symptoms following the stimulation.

During stimulation, more than half of the participants (60%) reported a mild sensation of dizziness, which disappeared immediately after the stimulation was turned off. No other sensory or postural effects were reported, except in one case, where mild nausea was reported, which, however, had already existed before the stimulation was started. Nearly all participants experienced a mild headache (90%) and/or a slight tingling sensation at the electrode placement sites on their skin (80%) (Figure 2). After adjusting the electrodes to restore optimal contact with the skin, this discomfort was diminished in most cases. None of the subjects reported pain at the stimulation site. The headache, often described as a pressing sensation near the temples, aligned with the position of the headband securing the electrodes. Six out of eight participants wearing the headband only as a control reported similar discomfort. After the stimulation ended and the electrodes were removed, no lingering symptoms were reported that had not been present prior to the stimulation.

## 4 Vestibular nature of stimulation

After evaluating the compatibility of long-term stimulation, we closely examined the core function of the mobile stimulation device. Specifically, we assessed whether it effectively stimulates vestibular pathways at suprathreshold intensity and elicits vestibular sensorimotor reflex responses as intended. We stimulated 6 healthy individuals (2 females; mean age 30.7 ± 4.4 years) with the maximum available noise intensity (peak amplitude of ±1 mA, 360 s stimulation duration). Simultaneously, we recorded body sway with an inertial measurement unit (IMU, Xsens, Movella Technologies, Enschede, The Netherlands) attached to the lower back (sacrum area) to assess vestibulospinal reflex responses. Since bipolar GVS stimulation primarily elicits a reflex response in the roll plane, the analysis focused on vestibulospinal responses in this plane (IMU-derived Euler angle in roll plane).

Correlation analysis in the frequency domain (magnitude-squared coherence) was performed to estimate average stimulation-induced variations in postural sway (39, 40). Coherence estimates with 95% confidence limits were calculated from the auto-spectra of the stimulation, body sway movement signals, as well as their cross-spectrum, using a finite fast Fourier transform with a block size of 1.28 s, providing a frequency resolution of 0.78 Hz (95% confidence limit for coherence estimates of 0.16 × 10^−3^). Coherence is a unitless measure between 1 (perfect linear relationship) and 0 (independence of signals).

The analysis (Figure 1C) demonstrated that the stimulation elicits significant vestibulospinal responses in the frequency domain (with peak coherence around 3 Hz). These findings confirm that the mobile stimulator effectively activates established vestibular sensorimotor pathways at suprathreshold stimulation levels.

## 5 Faciliatory effects of stimulation

**Figure 3:**
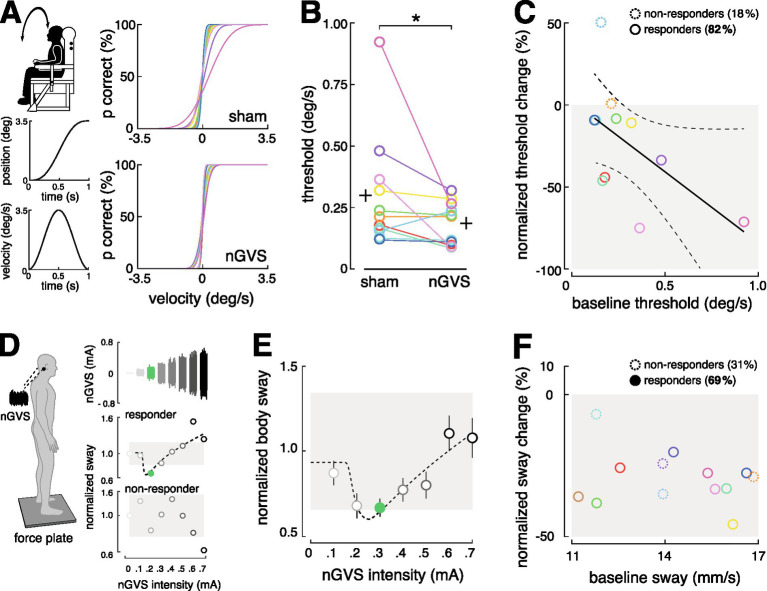
Therapeutic effects of stimulation. **(A)** The effect of nGVS stimulation on vestibular perception in the roll plane was examined using a psychophysical paradigm on a motion platform. Individual psychometric curves for perceptual performance under zero-current sham vs. nGVS stimulation were calculated. **(B)** The nGVS stimulation led to an effective reduction in the perceptual threshold in 82% of the participants. **(C)** Participants with poorer baseline performance were more likely to benefit from the stimulation. **(D)** The effect of nGVS stimulation on postural control were assessed on a force plate. Participants who showed reduced body sway at intermediate nGVS intensities, and no effect or increased body sway at low or high intensities, were classified as responders (69%); the others were classified as non-responders (see exemplary participant outcomes). **(E)** Average modulation of body sway from all responding participants. **(F)** No correlation was observed between baseline performance and the response or non-response to the stimulation. nGVS, noisy galvanic vestibular stimulation.

After confirming that the mobile stimulator activates vestibular pathways, we further investigated its potential therapeutic function. Specifically, whether subthreshold noise stimulation with the new device can reproduce facilitatory effects from the literature on vestibular perception thresholds and static postural control. Starting with perception, we investigated in 11 healthy young adults (5 females; mean age 29.0 ± 4 years) whether the stimulation effectively lowers the vestibular perceptual threshold, as previously reported (23–25, 28, 35). Using an established psychophysical paradigm, vestibular perception thresholds were determined as direction recognition thresholds (DRT) for head-centered roll-tilt motion (28). The DRTs were measured on a 6DOF motion platform (Moog 6DOF2000E, East Aurora, New York) two times with subjects either receiving zero-current sham stimulation (0 mA) or nGVS at a fixed intensity of 0.3 mA, in randomized order. During the investigation, non-vestibular cues were minimized using noise-canceling headphones and complete darkness (Figure 3A).

Stimulation effectively reduced the perceptual threshold in 82% of participants (Wilcoxon signed-rank test, *p* = 0.032). Consistent with previous findings, individuals with a higher (i.e., worse) baseline perceptual threshold showed a greater likelihood of benefiting from stimulation (Spearman correlation coefficient *R* = 0.618; *p* = 0.049) (24, 28) (Figures 3B,C).

Effects on postural control were investigated in 13 healthy participants (5 females; mean age 28.6 ± 4.0 years) using a force platform (Kistler, 9261A, Kistler Group, Winterthur, Switzerland, 40 Hz), following a previously established experimental paradigm. Participants stood quietly on the platform with their eyes closed for 30 s in eight trials, while nGVS of varying intensities, from 0 mA (sham) to 0.7 mA, was applied in a randomized order. For each trial, the mean velocity of the center-of-pressure displacement was computed. The presence of a stabilizing effect on postural control was evaluated by assessing the change of body sway across the range of applied nGVS intensities, as previously described (32, 34, 41, 42) (Figure 3D). A positive response would be indicated by a reduction in body sway at medium nGVS intensities, no change at low or high intensities, or an increase at higher intensities, resulting in a characteristic bell-shaped response curve typical of SR effects.

Three experienced human evaluators rated whether these criteria were met for each participant. The evaluation showed that stimulation with the mobile stimulator led to a stabilization of postural control in 69% of participants (Figures 3D,E). In contrast to previous reports in patients, our healthy participants did not exhibit any correlation between baseline body sway and the response to nGVS stimulation (Figure 3F).

## 6 Discussion

Non-invasive low-intensity vestibular noise stimulation (i.e., nGVS) is a promising method to enhance vestibular function and improve postural stability in vestibular hypofunction. Recent research evidence indicates that for this treatment approach to be effective, it must be applied continuously in patients’ daily routines (35, 36, 43). To facilitate the translation of nGVS therapy into everyday real-world applications, we introduce a novel mobile, wearable nGVS device. The prototype device is equipped with comprehensive safety features – including automatic shut-off, overload protection, and gradual adjustments in stimulation intensity to prevent abrupt changes that could cause discomfort or injury. These mechanisms have been tested for safety and tolerability in human use.

In a series of experiments, we evaluated the device’s function to activate the vestibular periphery and to elicit facilitatory effects on the vestibular perceptual and balance control level. Our mobile stimulator uses an AC power source for energy efficiency. Unlike stationary DC stimulators, the noise signal generated by the new stimulator has a slightly higher lower frequency cut-off at around 0.5 Hz (compared to commonly reported 0.02 Hz (43–46)). Irrespective of this difference, our investigations demonstrate that the mobile stimulator can reliably elicit stable vestibular reflex responses (39, 40) and reproduce faciliatory effects on vestibular perception and postural control, comparable to those observed in previous studies with stationary DC stimulators (23–26, 35, 42, 47). Taken together, these findings indicate that the new device performs its intended function effectively.

nGVS therapy is required only when the patient is active and moving, not during periods of rest or sleep. To accommodate this, the mobile nGVS device is equipped with a motion sensor that automatically activates or deactivates stimulation based on the user’s activity level. This feature not only improves the device’s energy efficiency but also provides additional advantages. Previous studies have demonstrated that a motion sensor placed on the head can not only differentiate between active and inactive phases but also provide detailed insights into daily mobility patterns, such as sitting, standing, walking, stair climbing, and even specific gait characteristics (48–50). In the future, such information could offer valuable feedback on the therapy’s effectiveness in improving mobility and reducing fall risk. The mobile nGVS device further features a user-friendly interface that allows easy adjustment of stimulation parameters for individual use and could be operated effortlessly by participants in our initial studies.

Although our mobile nGVS stimulator is compact and portable, it is currently limited using large conventional electrodes, which are unsuitable for daily use. In initial evaluation studies, we used electrode gel and secured the electrodes behind the ear with an elastic headband to improve conductivity. However, electrode displacement from head movements and pressure from the headband likely contribute to the commonly reported discomfort and long-term side effects, such as skin irritation and tension headaches. Similar side effects have been previously reported in other studies involving prolonged application nGVS (51–54), while no such symptoms were described during short-term stimulation (38, 53, 55).

For the device’s success in clinical use, these side effects must be addressed through further innovations in stimulation electrode design. Previous results indicate that the therapeutic effects of nGVS are likely enhanced by more focal stimulation (i.e., smaller electrodes) (56, 57), which would allow to effectively reduce the skin-electrode contact area. To prevent tension headaches, downsized electrodes must be further securely placed behind the ear. Stable skin contact needs to be ensured without the use of a headband or similar accessory, as demonstrated for instance by bone-conducting headphones that can be worn comfortably for extended periods without side effects (58). Furthermore, different electrode materials (54, 59, 60) and, if necessary, conductive media should be evaluated for both functionality – including their ability to maintain low impedance, durability, and consistent performance over extended use – and user factors such as comfort, reusability, and adaptability to bone contours (60–64). In addition to optimizing the device’s electrodes, studies in clinical cohorts are necessary to assess its long-term viability and effectiveness of the mobile nGVS device.

In conclusion, this study presents a new mobile nGVS device that adheres to strict safety standards and successfully replicates the facilitatory effects known from stationary nGVS devices. Further optimization of the stimulation electrodes is essential to ensure practicality and tolerability for everyday use. Once these challenges are addressed, the device will allow the treatment approach to be integrated into daily life for the first time, enabling a more precise evaluation of its therapeutic effects on clinically relevant outcomes, such as mobility, gait, and fall risk. Overall, the introduction of the mobile nGVS prototype device represents an important first step toward establishing a broadly available therapeutic tool for patients with chronic vestibular hypofunction and related disorders.

## Data Availability

The raw data supporting the conclusions of this article will be made available by the authors, without undue reservation.
